# Progress and prospects of medical education research in Asian Countries

**DOI:** 10.12669/pjms.35.6.1147

**Published:** 2019

**Authors:** Sultan Ayoub Meo, Kamran Sattar, Chaudhary Habib ullah, Sami Alnassar, Waseem Hajjar, Adnan Mahmood Usmani

**Affiliations:** 1 Sultan Ayoub Meo, MBBS, PhD. Department of Physiology, College of Medicine, King Saud University, Riyadh, Saudi Arabia; 2 Kamran Sattar, MBBS, M Med Ed. Medical Education, College of Medicine, King Saud University, Riyadh, Saudi Arabia; 3 Chaudhary Habib ullah, MBBS, FCPS. Orthopedic Surgery, College of Medicine, King Saud University, Riyadh, Saudi Arabia; 4 Sami Alnassar, MD, FRCS. Thoracic Surgery, College of Medicine, King Saud University, Riyadh, Saudi Arabia; 5 Waseem Hajjar, MD, FRCS. Thoracic Surgery, College of Medicine, King Saud University, Riyadh, Saudi Arabia; 6 Adnan Mahmood Usmani, MA, MCSE. University Diabetes Centre, College of Medicine, King Saud University, Riyadh, Saudi Arabia

**Keywords:** Asia, Medical education, Indexed journal, Research

## Abstract

**Background and Objectives:**

Medical education has a profound impact on health care system. Progress in achieving medical education research goals varies over time and across countries. This study aimed to investigate the medical education research ambience in Asia during the period 1965-2015.

**Methods:**

We investigated the bibliometric indicators of 49 Asian states in medical education research from 1965-2015. The data about Asian countries, their per capita GDP, expenditure on R&D, universities and indexed scientific journals were collected. We recorded medical education related research documents published in Institute of Scientific Information (ISI) Web of Science, Thomson Reuters during the period 1965-2015.

**Results:**

Asian countries collectively published 12721799 research articles, among them 40628 (0.31%) publications were in medical education. China contributed total of 3351565 articles among which 5414 (0.16%) research articles were in medical education; India added 1328725 papers with 4563 (0.34%) in medical education; Japan produced 3080257 papers with 4199 (0.13%) in medical education; Israel 561531 with 3848 (0.68%) in medical education; and lastly, Georgia contributed a total of 296532 research articles with 2565 (0.86%) in medical education.

**Conclusions:**

In Asia, the top five countries in medical education research are China, Georgia, Israel, Japan and India. The countries at low ranking are Yemen, Palestine, Myanmar, Kazakhstan, Syria and Armenia. In Asian states, the overall performance in medical science research needs policies to enhance its impact globally. Medical universities should offer research programs for learning and understanding the challengeable issues in medical education research.

## INTRODUCTION

Asia is the largest and most populous continent on the globe with 4.40 billion people.^1^ Asia is marked *by its history*, multi-cultural traditions, *geography, and vast social, economic and political diversity*.^1^ The pattern of health & disease, and health care system in Asia is very diverse.^2^

A few *Asian states* are swiftly stirring to uphold a quality medical education and are giving ardent extraordinary consideration to nurturing higher education and research in the region.^3^ Medical education has a broader impact on medical teaching and clinical practice. The research in medical education and associated health sciences has a noteworthy contribution to the nation’s monetary development with long-standing sustainable growth, and, ultimately, upgrading the health care system and living standard.^4^ To quantify the research progress of any nation, bibliometric measures including research documents and their visibility in global science are primary tools to comprehend growth and global extent.^5^

The research documents are strategic gauges for the development of nations and provide foundation for planned decisions for social and economic success.^6^,^7^ Asian states are fostering the promotion of education and research culture in the region. However, there is an acute lack of medical education research in the region. Therefore, the present study aimed to investigate the medical education research ambience in Asia during the period 1965-2015.

## METHODS

For the present study, we reviewed the data base literature in the month of June 2018 on “medical education” theme in Asian countries. We examined 49 Asian countries listed as per United Nations.^8^ The data about Asian countries, their per capita GDP, expenditure on research and development (R&D), universities and indexed scientific journals were recorded. Medical education allied studies on health professions exploring various aspects in teaching, learning, curriculum, admissions and assessment were collected. The articles including original articles, reviews, editorials, letters to editors published in Institute of Scientific Information (ISI) Web of Science, Thomson Reuters during the period 1965-2015 were recorded and analyzed.^9^ We excluded 10 Asian countries, which produced less than 25 research articles in medical education during the period 1965-2015, and finally, 39 Asian states were selected.

The information about the number of universities was recorded from the World Association of Universities.^10^ The details about medical and allied health science schools were recorded from websites of the concerned country and licensed organizations. We used the Web of Science for evaluating the ISI indexed journals. The country was selected, the individual Asian state appellation e.g. “China, Saudi Arabia, Pakistan, India, Israel, Malaysia” etc, was entered and the information of detailed journals along with the impact factors for each journal was retrieved from the Journal Citation Report, Institute of Scientific Information (ISI) Web of Science, Thomson Reuters.^9^ For the recording of research documents in “medical education” in global scientific journals indexed in ISI Web of Science, a particular country was selected, subject field “Medical Education” was entered and comprehensive information about number of research papers, total citations, citations per document^9^ and Hirsch Index (*h*-index) in medical education was obtained.

### Ethical statement

Ethical approval was not obtained as humans or animals were not involved. We acquired data based literature on medical education in Asian countries.

### Statistical Analysis

Statistical Package for the Social Sciences (SPSS) Inc Chicago, IL, USA, software version 21 was used to analyze the data. The results were presented as Mean±Standard Error of Mean (SEM). The Pearson correlation coefficient was used to find the strength of relation between different variables. *p*-value <0.05 was considered significant.

## RESULTS

In Asian states, the total number of universities and degree awarding institutes are 3444 with an average number of 88.30 per country. There are a total 1051 ISI indexed scientific journals (mean 26.95). The sum of their GDP is $509120.98 (mean $13054.38) and the mean R&D spending of Asian countries is 0.67% (Table-I).

**Table I:** Number of universities, medical schools, ISI Indexed journals, GDP and spending on R&D of Asian countries during the period 1965-2015.

Country	Number of Universities	Number of Medical Schools	Number of ISI Indexed journals	GDP	R&D
Georgia	13	12	1	2699	0.2
Israel	22	5	12	27341	4.14
China	389	132	173	3938	1.42
Japan	567	85	235	40101	3.28
India	367	346	100	1215	0.8
Taiwan	86	12	34	32316	2.33
South Korea	146	42	103	20108	3
Turkey	92	78	53	9729	0.7
Lebanon	24	5	0	8146	0.3
Singapore	6	3	52	38935	2.12
Thailand	67	18	8	4211	0.26
Bangladesh	80	70	4	608	0.4
Pakistan	189	156	12	1002	0.41
Iran	197	60	44	4403	0.62
Malaysia	146	26	10	7987	0.82
Russia	316	83	147	12736	1.14
Saudi Arabia	64	30	10	16862	0.13
Philippines	117	44	4	1991	0.12
Vietnam	46	13	0	1136	0.18
Indonesia	181	74	0	2550	0.1
Jordan	30	5	1	3976	0.39
Kuwait	8	1	5	50567	0.13
Nepal	8	21	1	478	0.21
UAE	36	6	38	44544	0.49
Qatar	2	2	0	75176	0.47
Sri Lanka	25	9	1	2184	0.15
Cambodia	20	3	0	764	0.1
Bahrain	12	4	1	18868	0.04
Cyprus	16	5	0	29733	0.37
Laos	2	1	0	1661	0.04
Iraq	48	23	0	2582	0.04
Mongolia	10	7	0	2144	0.25
Oman	11	2	0	20524	0.17
Yemen	14	6	0	1181	0.11
Myanmar (B)	34	13	0	1204	0.12
Palestine	16	5	0	1194	0.1
Kazakhstan	28	8	0	8553	0.21
Syria	15	7	0	2590	0.2
Armenia	12	5	3	3184	0.24
Total	3444	1365	1051	509120.98	26.3
Mean	88.30	35	26.94	13054.38	0.67
SEM	20.28	9.79	8.68	2772.44	0.12

The data were recorded from ISI-web of Science, 2017.^9^

***Note:*** GDP and R&D spending is presented in a mean.

The total number of research articles published from Asian countries in ISI indexed journals during the period 1965-2015 are 12721799 (mean 326199.97), total number of publications in medical education are 40628 (mean 1041.74), citations 438634 (mean 11247.02), citations per documents 9.39, Hirsch Index (*h*-index) 1337 (mean 34.30) (Table-II).

**Table II:** Number of research documents published in Medical Education in ISI web of science, total citations, citations per document, Herish Index and overall ranking of Asian countries in Medical Education during the period 1965-2015.

Country	Total publications	Total Medical Education publications and %	Total Citations	Citations per Document	H Index based on Medical Education	Country Ranking in Medical Education
Georgia	296532	2565 (0.86%)	52874	20.61	101	1
Israel	561531	3848 (0.68%)	66932	17.39	98	2
China	3351565	5414 (0.16%)	61384	11.34	89	3
Japan	3080257	4199 (0.13%)	42541	10.13	81	4
India	1328725	4563 (0.34%)	39906	8.75	76	5
Taiwan	532496	2044 (0.38%)	20494	10	57	6
South Korea	812407	1832 (0.22%)	17932	10	55	7
Turkey	467872	2403 (0.51%)	19016	8	52	8
Lebanon	44953	750 (1.66%)	12991	17	51	9
Singapore	215698	1514 (0.70%)	13658	9	49	10
Thailand	127908	1256 (0.98%)	12576	10	49	11
Bangladesh	30447	467 (1.53%)	8471	18.14	41	12
Pakistan	89891	1043 (1.16%)	9740	9.34	37	13
Iran	322322	1971 (0.61%)	9261	4.7	35	14
Malaysia	150331	946 (0.62%)	6258	6.62	34	15
Russia	861873	1111 (0.12%)	5052	5	34	16
Saudi Arabia	126521	1450 (1.14%)	7394	5	32	17
Philippines	26588	271 (1.01%)	4906	18	31	18
Vietnam	30571	245 (0.80%)	2701	11	29	19
Indonesia	39225	334 (0.87)	3030	8.81	27	20
Jordan	32798	278 (0.84%)	4583	16	27	21
Kuwait	22570	185 (0.81%)	2341	13	26	22
Nepal	10776	354 (3.28%)	3082	9	26	23
UAE	15017	223 (1.55%)	2180	10	25	24
Sri Lanka	15400	285 (1.85%)	1618	6	18	25
Qatar	14505	180 (1.24%)	1150	6	18	26
Cambodia	2671	8 (3.10%)	804	9.69	16	27
Bahrain	4486	123 (2.74%)	913	7	15	28
Cyprus	18466	108 (0.58%)	1111	10.29	14	29
Laos	1720	58 (0.04%)	645	11.12	14	30
Iraq	14317	83 (0.57%)	740	8.92	13	31
Mongolia	14081	48 (0.34%)	356	7	12	32
Oman	11855	134 (1.13%)	807	6	12	33
Yemen	2913	58 (1.99%)	378	7	11	34
Palestine	2054	34 (1.65%)	257	8	8	35
Myanmar	1475	34 (2.30%)	171	5.03	8	36
Kazakhstan	15167	60 (0.39%)	133	2.22	7	37
Syria	6087	49 (0.80%)	199	4	7	38
Armenia	17728	25 (0.14%)	49	1.764	3	39
Total	12721799	40628 (0.31%)	438634	366.414	1337	N/A
Mean	326199.97	1041.74 (0.31%)	11247.02	9.39	34.30	N/A
SEM	120092.56	210.63	2123.68	0.625	3.40	

The data were recorded from ISI-web of Science, 2017.^9^

**Fig. 1:**
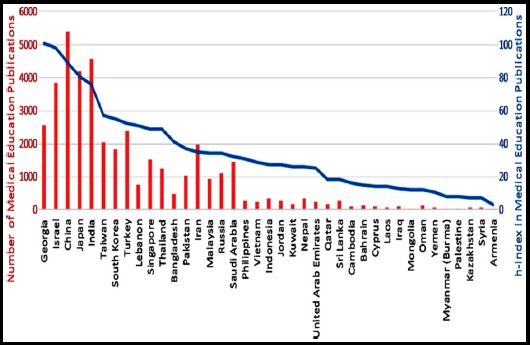
Total number of publications in medical education in ISI web of science and Herish Index of Asian countries in medical education during the period 1965-2015.

**Fig. 2:**
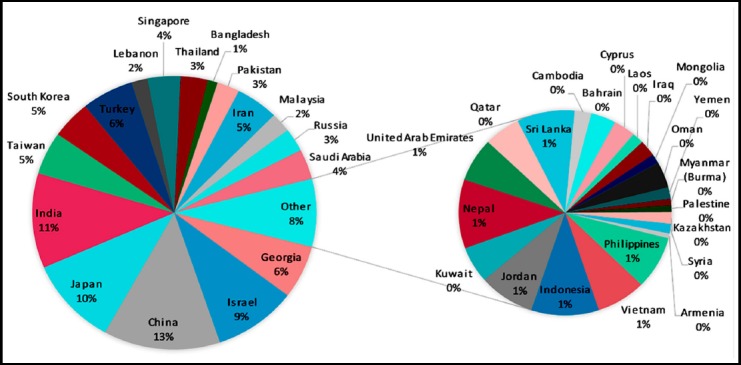
Asian countries contribution in medical education research published in ISI web of science during the period 1965-2015.

Based on research documents published in medical education in ISI-Web of science, the top five Asian countries are China contributing 5414, India contributing 4563, Japan 4199, Israel 3848 and Georgia with 2565 research papers in Medical Education (Fig. 1). However, based on Hirsch Index (*h*-Index) in medical education, the ranking of Asian countries is: Georgia (101), Israel (98), China (89), Japan (81) and India (76) (Fig. 1). While considering the percentage of medical education documents in total research documents in a particular country, Georgia had 0.86%, Israel 0.68%, India 0.34%, China 0.16% and Japan 0.13% (Table-II). The percentage of medical education documents in comparison to total number of medical education research documents in entire Asia was: China 13%, India 11%, Japan 10%, Israel 9%, Georgia and Turkey 6% each (Fig. 2).

**Fig. 3:**
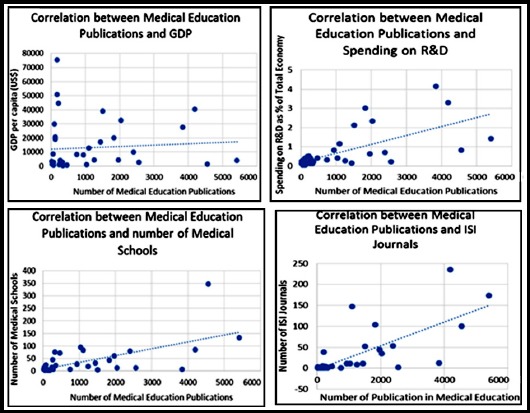
Correlation between number of publications in medical education with GDP, spending on R&D, number of medical schools and ISI indexed journals.

We established the Pearson correlation coefficient and a positive association was observed between medical schools and total number of research articles appearing in ISI Web of Science (r^2^=0.458, *p*=0.003). Similar observation was also found between the number of medical schools and research appearing in the area of medical education (r^2^=0.541, *p*= 0.0001) (Fig. 3).

## DISCUSSION

Medical education research has a profound impact on the health care system.^3^ In Asia, the top five countries in medical education research are China, Georgia, Israel, Japan and India. On the other hand, countries at low ranking are Yemen, Palestine, Myanmar, Kazakhstan, Syria and Armenia. In Asian countries, the medical education system shows marked diversity depending on the social, cultural and educational methods. Few Asian countries are trying to enhance research in medical education.^3^,^11^

Majumder^12^ reported that medical education in Asia is not a need-based education, since bureaucrats under the influence of international donor agencies make major decisions and most of the decisions are based on political and financial interest, deliberately ignoring the relevance of medical education research. The present study findings agree with Majumder^12^ that in Asian countries currently there are 3444 universities and among them, 1365 are medical universities (Table-I), but not a single one of them flourished and achieved a standing like US or European universities to be a flagship medical education institute.

As per Shanghai ranking of the world universities 2017^13^, America has 16 and Europe has four universities among the top 20 universities. However, none of the Asian universities achieved the ranking among the top 20 universities.^13^ Moreover, among top 100 universities, America has 52 universities; Europe has 35; and Asia has 13 universities in medical and health sciences.^13^ This reflects the poor performance of Asian universities in global research production.

**Fig. 4:**
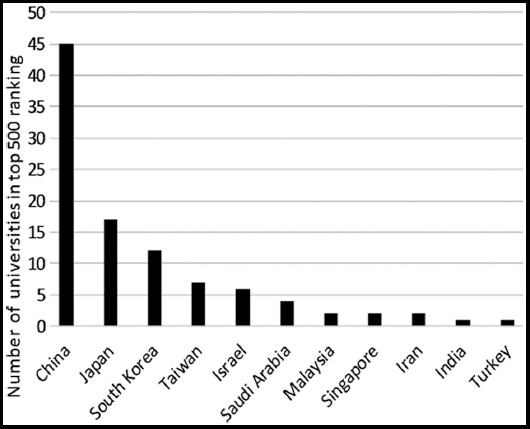
Top 500 universities in Asian countries (Academic Ranking of World Universities Shanghai Ranking Report-2017)^13^

In the present study, we found that very few Asian states have produced research in medical education. Asian countries such as China, Georgia, Japan and Israel have developed educational policies and collaborated with highly reputable institutes of postgraduate excellence. Israel increased emphasis on education and research in both basic and clinical medical sciences and education. The medical educational tools are interactive and learner-centered rather than being frontal teaching formats.^14^ In Israel, there are 22 universities, 12 medical schools, and it has 12 ISI Indexed journals (Table-I). As per Shanghai Ranking 2017^13^, Israel has five universities and degree awarding institutes within the top 500 universities (Fig. 4). Israel produced 561531 research articles and among them, 3848 research articles are in medical education with h-index of 98. As per World Bank report, Israel is spending 4.41% of their GDP on R&D which is the highest in the world^15^ and has highly ranked universities. These are the main reasons that Israel is ranked 2^nd^ in Asian states in medical education research (Table I and II).

China has a population of approximately 1.3 billion, occupying a huge diverse landmass and making a rapid transition from a Third-World to a First-World economy.^16^ China has 389 universities, 132 medical schools, 173 ISI indexed journals, and is spending 1.42% of their GDP on R&D (Table-I). It also has 45 Universities among the top 500 universities.^13^ China is contributing a significant amount of research articles in ISI Web of Science (3351565), medical education (5414), h-index (89) and is currently holding third place in research in medical education among the Asian countries (Table-II).

China has been moving fast, funding enormously with a swift growing science skilled personnel. However, Chinese research has to develop strategies to further enhance its impact, which the country is trying to address. China is trying to increase research funding to 2.5% of the country’s GDP by 2020. One area in which the country is competing globally is molecular medicine, and desires to dominate precision medicine. The world is observing China racing forward, but it still has a long way to go before it becomes a leader in medical education research and innovation.^16^

India has 367 universities, 346 medical schools, 100 ISI indexed journals, and has published 1328725 research articles, among which 4563 are in medical education with h-index of 76. As per our study findings, among Asian countries, India is standing among the top five countries in medical education. Despite its huge size and population of about 1.3 billion people, the number of universities and research institutes and their outcome has not been satisfactory. Indian universities and their research are mired in bureaucracy and insufficient funding, being less than 0.9% of the GDP since 2005. All these factors have muted the spirit of academic excellence and hampered research^17^ especially in medical education. The other Asian countries attempting to enhance research in medical education are Taiwan, South Korea, Turkey, Malaysia, Pakistan, and oil rich Gulf Cooperation Council (GCC) states.

In Pakistan, there is an increasing appreciation towards establishing medical education departments and postgraduate degree programs in medical and dental schools. However, the infrastructure, availability of trained faculty and financial resources are serious issues. Majority of the medical schools do not have a full time qualified medical education faculty. The Higher Education Commission (HEC) and Pakistan Medical and Dental Council (PMDC) should provide policy to strengthen the medical education and its allied research in the country.^18^

The GCC countries are constantly increasing their spending on education and research funding. The educational budget of Saudi Arabia reached $54.54 billion for the year 2015.^19^ GCC universities are trying to establish an international alliance to promote research culture. However, the research outcome in medical education from GCC states is still marginalized.^3^

Hamdy et al.^20^ reported that in Asia, GCC states are in a major social, cultural and fiscal transformation. However, in the region, the progress of medical education is relatively newfangled. Medical schools’ curricula are variable without integration, and there are faculty shortages, lack of training facilities and faculty development in medical education. In concurrent to the findings of Hamdy et al.^20^, we believe that these are the reasons that in Asian states, the wealthy GCC nations have not produced a significant volume of research in medical education.

In another study, Khalid BA^21^ demonstrated that in GCC states, medical universities are changing their curricula to maintain efficiency. Many medical schools followed the traditional curriculum, while a few followed a hybrid problem-based learning (PBL) curricula. Even with the curriculum transformation, GCC states produced little research in medical education. In another study, Khalid Abdulrehman^22^ reported that several factors obstruct medical education research in the region including lack of medical education research funding, absence of biomedical industries interest in medical education research, lack of research skills, and dearth of regional peer-reviewed ISI indexed journals in medical education.

Choung and Hwang^23^ reported that universities play an essential role in increasing number of research articles in the ISI database. In Asian countries, the total numbers of universities are 3444, from which 365 are medical universities or colleges. In the present study, we found that since the number of universities increased, number of research papers also increased. Meo et al.^24^ found that states with a vast number of universities produced more research articles. Congruently, in this study, we found that Asian states which increased the number of universities showed better research outcome in medical education.

### Limitations of the study

The strengths of this study are that we searched the research performance of Asian countries from very reliable sources which include Institute of Scientific Information (ISI) Web of Science, Journal Citation Reports (Thomson Reuters) and World Bank. A comprehensive search of the literature would undoubtedly enrich the framework of available strategies for identification of ambience. However, our study had a few limitations. Occasionally, citation count tools may mis-cite or re-cite a paper, which may inflate the number of documents or citation sums. We also excluded 10 Asian countries that produced less than 25 research articles in medical education.

## CONCLUSION

In Asia, the top five countries in medical education research are China, Georgia, Israel, Japan and India. However, the countries at the bottom line ranking are Yemen, Palestine, Myanmar, Kazakhstan, Syria and Armenia. A multidimensional approach is required to improve medical education research in Asian countries, and international alliances with universities and research rich institutes should be established to enhance research and its visibility.

### Recommendations

Medical universities should offer compulsory research programs and research elective for their graduation which would provide the foundation for a durable approach to learning and understanding the challengeable issues in medical education research.

### Authors’ Contribution:

**SAM** contributed to the study design, literature review, analysis and interpretation of data and writing the manuscript. Takes responsibility of integrity of research.

**KS, CH, SA, WH and AMU** were involved in literature review and data analysis.

All authors have read and approved the manuscript.
